# Autophagy in aquatic animals: mechanisms, implications, and future directions

**DOI:** 10.3389/fimmu.2025.1612178

**Published:** 2025-06-03

**Authors:** Md. Abu Kawsar, Diponkor Adikari, Yang Zhang

**Affiliations:** ^1^ State Key Laboratory of Breeding Biotechnology and Sustainable Aquaculture, Guangdong Provincial Key Laboratory of Applied Marine Biology, South China Sea Institute of Oceanology, Chinese Academy of Sciences, Guangzhou, China; ^2^ Sanya National Marine Ecosystem Research Station, Tropical Marine Biological Research Station in Hainan, Key Laboratory of Tropical Marine Biotechnology of Hainan Province, Chinese Academy of Sciences, Sanya, China; ^3^ University of Chinese Academy of Sciences, Beijing, China; ^4^ Department of Aquaculture, Sylhet Agricultural University, Sylhet, Bangladesh; ^5^ Department of Aquatic Resource Management, Sylhet Agricultural University, Sylhet, Bangladesh

**Keywords:** autophagy, autophagy-related gene (ATG), aquatic animal health, host-pathogen interactions, lysosome-autophagy system

## Abstract

Autophagy, a highly conserved intracellular degradation process, is essential for maintaining cellular homeostasis, supporting development, modulating immune responses, and enhancing stress adaptation in eukaryotic organisms. In aquatic animals, growing evidence highlights the central role of autophagy in responding to diverse environmental stressors and microbial challenges-factors critical to aquaculture productivity and ecosystem health. This review synthesizes current knowledge on the regulation and function of autophagy in aquatic species, emphasizing key molecular pathways, environmental triggers such as temperature, salinity, hypoxia, and pollutants, and host responses to pathogenic infections. We explore model systems, particularly zebrafish, that have advanced our mechanistic understanding of autophagy, while also identifying gaps in research concerning economically important aquaculture species. Promising applications, including the use of autophagy modulators, probiotics, and gene-editing tools such as CRISPR/Cas9, are evaluated for their potential in disease prevention and environmental monitoring. Despite recent progress, selective autophagy pathways and species-specific regulatory mechanisms remain poorly understood. Future studies integrating high-throughput screening, multi-omics analyses, and functional genetics are essential to unlock the full potential of autophagy-based innovations for sustainable aquaculture development.

## 1 Introduction

Cell survival and homeostasis are fundamental biological processes that regulate development and immune defense. In aquatic animals, autophagy plays a crucial role in maintaining cellular integrity, responding to environmental stress, and enhancing resilience against infections (1, 2). Autophagy, often referred to as “self-eating,” is a highly conserved intracellular degradation process responsible for removing damaged organelles, misfolded proteins, and cytosolic components through lysosomal degradation, thereby maintaining cellular homeostasis (3, 4). This process ensures the complete and irreversible breakdown of substrates into their essential building blocks, such as amino acids from proteins and nucleotides to nucleic acids, through lysosomal enzymatic activity (3, 5). Beyond its role in energy balance under nutrient-deficient conditions, autophagy acts a critical defense strategy against germs and environmental stressors, contributing to the overall resilience and disease resistance of aquatic organisms. This self-digestive mechanism is essential for cellular survival, development, and stress responses across eukaryotes, from mammals to yeast (6). In recent decades, autophagy has gained attention due to its dual role in both pathological and physiological processes, including immunity, metabolism, and disease progression (7). While extensive research has elucidated the molecular mechanisms and regulatory pathways of autophagy in mammals, its functions in teleost fish and other aquatic animals remain relatively underexplored, despite their ecological and economic importance (8).

Although progress has been made, there are still considerable gaps in our knowledge of how autophagy is regulated in aquatic animals. The conservation of autophagy-related genes across species suggests shared mechanistic pathways, but species-specific adaptations and environmental influences warrant further investigation (9). Furthermore, the possible uses of autophagy modulation in aquaculture such as enhancing growth, boosting disease resistance, and increasing stress tolerance have not yet been fully explored or achieved. The aims of this review are to synthesize current knowledge on autophagy in aquatic animals, focusing on its mechanisms, monitoring methods, response to environmental stress, host-pathogen interactions, biotechnological implications, and future research directions. By bridging gaps between fundamental and applied science, this work seeks to advance our knowledge of autophagy and its role in aquatic biology and its applications for sustainable aquaculture practices.

## 2 Types of autophagy

**Figure 1:**
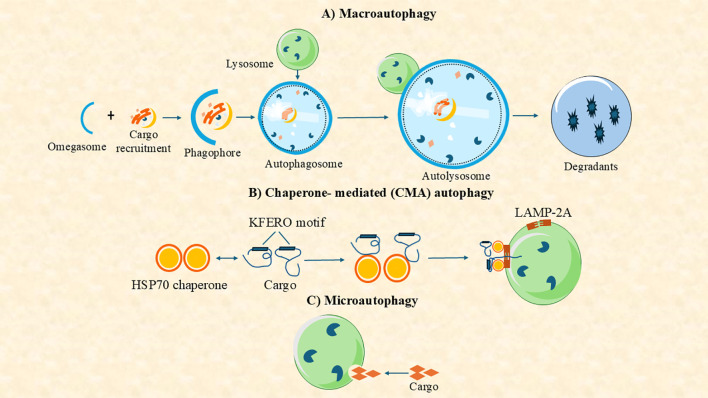
Autophagy can be classified into three main types based on the pathway used to deliver intracellular components (cargo) to the lysosome for degradation: **(A)** macroautophagy, **(B)** chaperone-mediated autophagy (CMA), and **(C)** microautophagy.

Autophagy is a well-conserved cellular mechanism found in all eukaryotic organisms, including aquatic species like fish, mollusks, and crustaceans (3, 10, 11). Depending on how substrates are delivered to the lysosomal lumen, three primary types of autophagy have been identified in mammalian cells: macroautophagy, microautophagy, and chaperone-mediated autophagy (CMA) (12) (**Figure 1**). Among them, macroautophagy is the most extensively studied in aquatic organisms, owing to its vital roles in cellular homeostasis, stress adaptation, and pathogen defense (13, 14). In this process, cytoplasmic materials are sequestered by a double-membrane structure called the autophagosome, which fuses with lysosomes for degradation (15–18). It can also be broadly classified into two major types found in nearly all eukaryotic organisms: canonical autophagy (macroautophagy) and non-canonical autophagy (**Figure 1**) (14). Macroautophagy has been widely investigated in aquatic species, especially in fish and shellfish, due to its relevance to aquaculture health and immunity.

Non-canonical forms of autophagy include microautophagy and CMA (**Figure 1**). Microautophagy involves the direct engulfment of cytosolic components by lysosomal membrane invagination or protrusion, bypassing autophagosome formation (19). While less studied in aquatic organisms, it has been implicated in nutrient recycling in mollusks and fish hepatocytes (20). CMA is a highly selective pathway in which proteins containing a KFERQ-like motif are recognized by the HSC70 chaperone and translocated into lysosomes via the LAMP2A receptor (21). Although well-characterized in mammals, CMA’s presence in aquatic animals remains uncertain. Nevertheless, CMA-related genes, such as LAMP2A, have been identified in zebrafish genomes (22). Overall, while macroautophagy is the most thoroughly characterized form in aquatic species, current research is beginning to elucidate the roles of microautophagy and CMA in aquatic physiology, nutrient regulation, and disease resistance.

## 3 Mechanism of autophagy

### 3.1 Molecular pathways

Autophagy is a tightly regulated process activated by stimuli such as pathogen invasion, nutrient deprivation, oxidative stress, and metabolic changes (10). The central regulator is the mammalian target of rapamycin (mTOR), a serine/threonine kinase that integrates various intracellular and extracellular signals to suppress or activate autophagy (23–26). mTOR forms two complexes-mTORC1 and mTORC2-with mTORC1 being modulated by growth factors, energy status, and stress via upstream regulators like AMPK, PI3K/AKT, and p53 (27–30). Additionally, kinases like PKR and ERK1/2, and factors such as eIF2α, also influence autophagic responses (31–33).

Autophagy is mediated by evolutionarily conserved autophagy-related (Atg) genes, first identified in yeast. Many of the >40 yeast Atg genes have homologs in mammals and aquatic species (34–39). Among them, 15 core Atg genes (e.g., ATG1–10, ATG12–14, ATG16, ATG18) are essential for autophagosome biogenesis (17, 40, 41). ULK1, the mammalian homolog of Atg1, forms a complex with ATG13, ATG101, and FIP200 to initiate autophagy, a step promoted by AMPK and inhibited by mTOR (41). This triggers recruitment of the PI3K complex (ATG14L/VPS34), producing PI3P that recruits WIPI proteins for phagophore formation (42–44).

**Figure 2:**
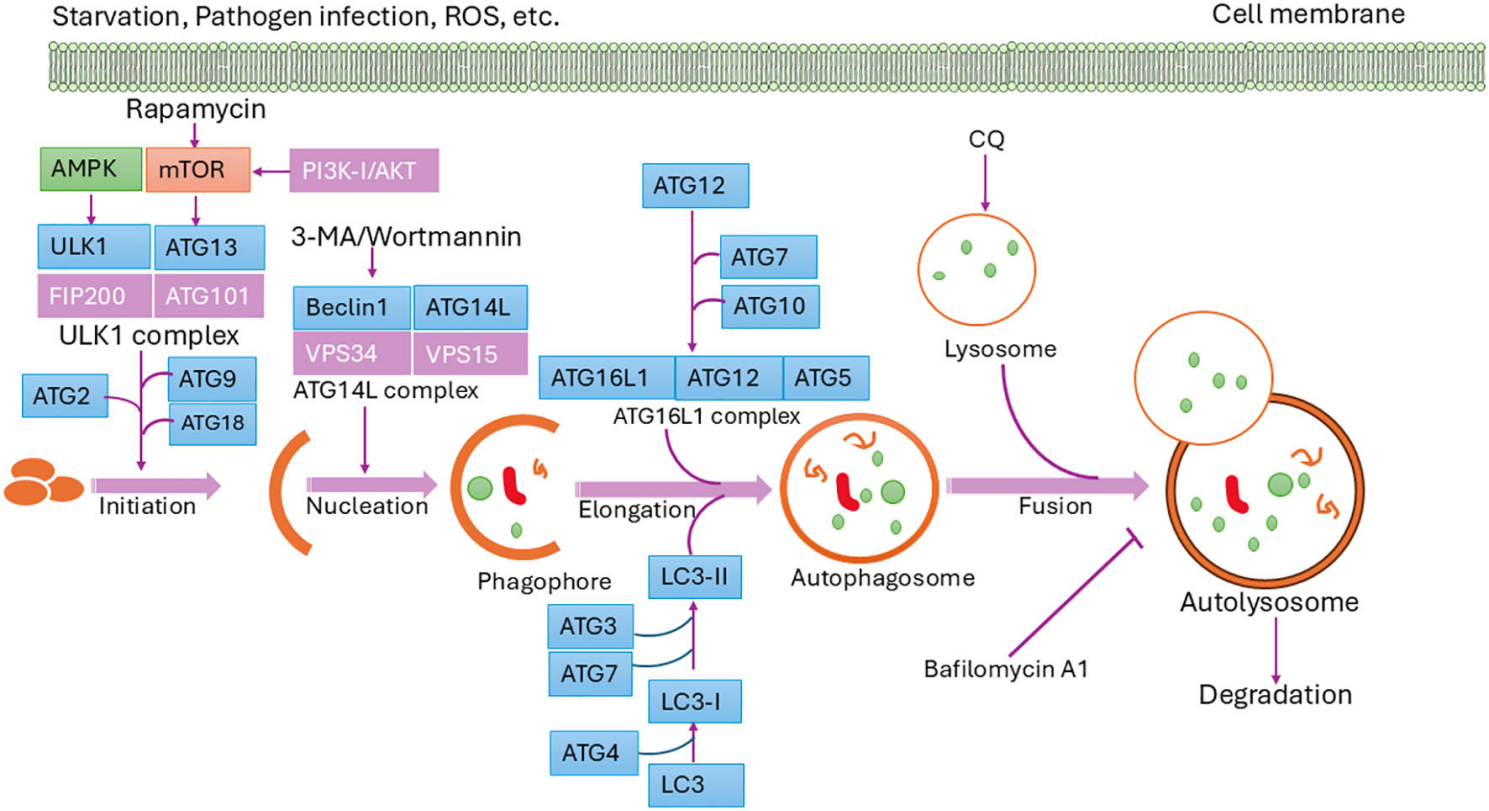
Schematic diagram of the autophagy pathway. Autophagy is initiated by specific signals, leading to the formation of the isolation membrane (phagophore), which matures into an autophagosome. Fusion with a lysosome forms an autolysosome, where degradation occurs. Core ATG proteins essential for autophagosome creation are highlighted in light blue. Donor membranes include the ER, Golgi apparatus, mitochondria, endosomes, and plasma membrane.

Elongation involves two ubiquitin-like conjugation systems. ATG12–ATG5–ATG16 and LC3 (Atg8 homolog) are central to autophagosome expansion. LC3 is processed by ATG4 to LC3-I, then lipidated to LC3-II by ATG7 and conjugated with PE (45, 46). Specific autophagy is mediated by cargo receptors (e.g., p62, NBR1, NDP52, optineurin) that link targets to LC3 via LIR motifs (41). Mature autophagosomes fuse with lysosomes, facilitated by SNARE proteins, cytoskeletal elements, and motor proteins, leading to cargo degradation and recycling of LC3 (17, 46). This process is vital for maintaining cellular homeostasis and adapting to environmental stress. These sequential steps are illustrated in **Figure 2**, emphasizing the dynamic regulation and cellular importance of autophagy (10).

Autophagy is not an isolated event but is intricately interconnected with several other key physiological processes that collectively maintain cellular homeostasis, including apoptosis, the unfolded protein response (UPR), and oxidative stress regulation (47–51). For instance, p53, a well-known tumor suppressor and upstream regulator of mTOR, plays a dual role by also governing apoptosis, thereby linking nutrient sensing, cell growth, and cell death pathways (47). Likewise, eIF2α, which contributes to autophagy initiation, is a central mediator of the PERK branch of the UPR, responding to endoplasmic reticulum stress and modulating downstream outcomes such as autophagy or apoptosis depending on severity and duration (49). Furthermore, reactive oxygen species (ROS), often elevated during oxidative stress, act as pivotal signaling molecules that can simultaneously activate autophagy, modulate apoptosis, and regulate the UPR through multiple pathways including PI3K/Akt, AMPK, JNK, ERK, and ATG4 (50, 51). These overlapping regulatory circuits underscore autophagy’s role as a central hub in the cellular stress response network, integrating diverse intracellular signals to balance survival and death decisions, beyond its classical role in nutrient sensing or pathogen clearance (34, 37).

### 3.2 Autophagy across taxonomic groups: evolutionary conservation and divergence

Autophagy is a conserved catabolic process across eukaryotes, though its regulation and physiological roles vary among taxa. In basal metazoans like Porifera and Cnidaria, genomic analyses reveal core autophagy-related genes (ATGs), suggesting ancient origins, though functional data remain limited (11). In Platyhelminthes, autophagy is essential for regeneration, as seen in planarians where it coordinates tissue remodeling (52). Insects exhibit autophagy during metamorphosis, indicating its developmental significance. While data in annelids are sparse, transcriptomic evidence supports autophagy’s role in regeneration and stress responses.

In arthropods, including crustaceans, autophagy contributes to immune defense and environmental adaptation. Among lophotrochozoans, mollusks offer detailed insights. Picot et al. (35) identified 35 ATG genes in *Crassostrea gigas*, showing widespread expression and homology with vertebrate counterparts, but also lineage-specific expansions likely linked to immune function and stress tolerance (35). Echinoderms also display expanded ATG families, possibly reflecting adaptation to regenerative demands. Moore et al. (11) highlighted autophagy’s role in toxicological responses in marine invertebrates, emphasizing its utility in environmental stress assessment (11). Overall, while autophagy’s core machinery is conserved, taxon-specific adaptations underscore its evolutionary diversification and multifunctionality, warranting further comparative studies in underrepresented invertebrate groups.

### 3.3 Autophagy in response to various stress

An overview of autophagy’s role in different aquatic species under various environmental stress conditions is provided in **Table 1**.

**Table 1:** Mechanisms of autophagy activation in response to environmental stressors.

Stress Factor	Species	Mechanism of Autophagy Activation	Key Genes Involved	Outcome & Transition to Apoptosis	Citation
Heavy Metal (Cd)	*Carassius gibelio*	Cd disrupts protein homeostasis, triggering ER stress and UPR pathways (PERK-eIF2α-ATF4, IRE1-XBP1), leading to autophagy activation.	beclin1, atg5, lc3b	Adaptive response initially; prolonged exposure leads to excessive autophagy and apoptosis (bax, casp3).	(53)
Chromium (Cr²^+^) exposure (oxidative stress)	*Channa punctatus*	Oxidative stress triggered autophagic response in liver and kidney	Upregulation of ATG5, LC3, GABARAP; downregulation of mTOR	Induced autophagosome formation, elevated ROS, oxidative stress markers, and micronuclei formation.	(54)
Copper (Cu²^+^) exposure	Chinese mitten crab (*Eriocheir sinensis*)	Oxidative stress and ER stress triggered autophagy via ERK and AMPK pathways	Autophagy-related genes, ERK, AMPK, TLR2-MyD88-NF-κB	Induced autophagy and apoptosis in hepatopancreas and gills; immune response activation through TLR2-MyD88-NF-κB pathway.	(55)
Hypoxia	*Ctenopharyngodon idella* (grass carp)	ROS accumulation activated autophagy via inhibition of Akt and activation of FoxO1	LC3-II, pink1, beclin-1, p62, foxO1a/1b, Hif-1α	Autophagosome formation observed; FoxO1 upregulation essential for hypoxia-induced autophagy	(55)
Pesticide (Fipronil)	Common carp (*Cyprinus carpio*)	Induces oxidative stress, activating the ubiquitination pathway (ATG5–ATG12–ATG16L) for autophagosome formation.	ATG5, ATG12, ATG16L, LC3-II, Beclin1	Initially protective, but prolonged exposure causes apoptosis (Bcl-2, Caspase-3).	(56)
Nitrite stress	grass carp (*Ctenopharyngodon idella*)	Nitrite exposure triggers ER stress, activating the GRP78 pathway, potentially inducing autophagy and apoptosis	GRP78, Ulk1, Beclin1, Atg5, Atg12, LC3, BNIP3, P62	Optimal dietary protein (22-25%) reduces nitrite-induced autophagy, alleviates gill damage, and inhibits apoptosis through mitochondrial and death receptor pathways.	(57)
Nutrient Deprivation (Serum Starvation)	Zebrafish (*Danio rerio*)	Serum deprivation activates autophagy through upregulation of autophagic genes (ulk1a, becn1, atg12, sqstm1, maplc3, lamp1), with initial boosting followed by weakening at 48 hours.	ulk1a, becn1, atg12, sqstm1, maplc3, lamp1	Autophagic activity initially compensates for stress; prolonged starvation leads to apoptosis (caspases, Bcl-2/Bax expression, Annexin V/PI).	(58)
Acute Ammonia Stress	Yellow catfish (*Pelteobagrus fulvidraco*)	Ammonia stress triggers autophagy via the SLC38A9-mTOR axis, initially inhibiting autophagy followed by restoration.	SLC38A9, LC3a, sqstm1	Inhibition followed by restoration of autophagic flux; excessive autophagy worsens ammonia toxicity, oxidative stress, and apoptosis.	(2)
Chronic Pollution (TFS)	Zebrafish (*Danio rerio*)	Trifloxystrobin (TFS) induces autophagy via mTOR inhibition, leading to an increase in autophagosomes and LC3-II conversion.	LC3-II, Beclin-1, P62, mTOR	Increased viral replication due to impaired immune response; TFS exposure may contribute to viral outbreaks.	(59)

#### 3.3.1 Starvation-induced autophagy

Starvation triggers a highly conserved autophagic response in teleost fish, facilitating cellular recycling and energy mobilization under nutrient-deprived conditions. In rainbow trout (*Oncorhynchus mykiss*), *in vivo* studies have shown that prolonged fasting induces autophagy in muscle and hepatic tissues, as indicated by the upregulation of autophagy-related genes and increased autophagosome formation, promoting protein degradation and nutrient redistribution (60, 61). Similarly, in adult zebrafish (*Danio rerio*), *in vivo* starvation for 1–3 weeks leads to systemic metabolic suppression, including decreased RNA/DNA ratios and impaired reproductive capacity, along with notable developmental disruptions in larvae such as delayed hatching and reduced survival (62). These effects are accompanied by significant transcriptional activation of autophagy-related genes in both maternal ovaries and offspring, indicating a maternal transference of starvation-induced autophagic signaling. Further transcriptomic analyses have shown that the zebrafish liver exhibits extensive downregulation of genes involved in lipid metabolism, proteolysis, and protein biosynthesis under starvation, while autophagy-related and gluconeogenic pathways are upregulated (63). In contrast, the zebrafish brain shows a limited response, with only agrp1 significantly upregulated, reflecting tissue-specific regulation of autophagy. Comparative analysis also reveals species-specific differences in hepatic transcriptional responses: while zebrafish and rainbow trout share similar starvation-induced expression patterns, common carp (*Cyprinus carpio*) displays a notably divergent profile (63), suggesting variable autophagy regulation across species. Although most findings are from *in-vivo* models, *in vitro* studies using fish-derived liver cell lines and primary hepatocytes-such as those from zebrafish-have also shown starvation-induced autophagy, including autophagosome formation and upregulation of key genes. These systems help clarify molecular mechanisms without systemic influences. In medaka (*Oryzias latipes*), early larval starvation induces hepatic lipid accumulation and shifts in amino acid and fatty acid metabolism, likely linked to autophagy (64).

Beyond fish, invertebrates also exhibit robust autophagic responses to starvation. In the bivalve mussel *Mytilus galloprovincialis*, starvation for nine days significantly upregulated autophagy-related genes such as atg2, atg6, and atg13, and increased autophagosome formation in gill tissues, suggesting a protective role of autophagy in response to nutritional stress (65). Similarly, the cnidarian *Hydra vulgaris* undergoes widespread autophagic vacuole formation in ectodermal epithelial cells during starvation, which is essential for survival; disruption of this process via RNAi-mediated knockdown of Kazal1 results in excessive autophagy and organismal death, underscoring the need for tightly regulated autophagic activity (66). Furthermore, evidence from annelids and planarians supports that autophagy plays a vital role in cellular maintenance during starvation across diverse aquatic invertebrates. These findings collectively highlight starvation-induced autophagy as a fundamental and evolutionarily conserved survival mechanism among aquatic taxa.

#### 3.3.2 Role of autophagy in heavy metal stress

Autophagy plays a critical role in mediating cellular responses to heavy metal exposure in aquatic organisms. As a protective mechanism, it helps mitigate oxidative stress and cellular damage induced by metals such as cadmium, lead, and mercury (67). However, chronic or excessive exposure can overwhelm autophagic capacity, leading to cytotoxic effects that impair fish health, growth, reproduction, and survival (68). Understanding the dual role of autophagy-both protective and potentially harmful- is essential for elucidating heavy metal toxicity pathways and for developing strategies to monitor and mitigate environmental contamination (67, 68). Below, we discuss how autophagy is modulated in response to exposure to different heavy metals in aquatic organisms.

##### 3.3.2.1 Autophagy in response to cadmium exposure

Autophagy is vital for cellular defense against environmental stressors like heavy metals and pollutants by preserving cellular homeostasis and eliminating damaged organelles and proteins. Exposure to heavy metals, such as cadmium (Cd), can trigger oxidative stress and endoplasmic reticulum (ER) stress, activating autophagy as a protective response in aquatic animals (53). Cd exposure disrupts protein homeostasis, triggering the unfolded protein response (UPR), which activates autophagy to reactive cellular balance (69). The PERK-eIF2α-ATF4 and IRE1-XBP1 of the UPR are critical in modulating autophagic responses to heavy metal-induced stress (70, 71). Studies in gibel carp (*Carassius gibelio*) suspected to waterborne Cd have shown significant upregulation of genes related to autophagy, including atg5, beclin1, and lc3b, suggesting a cellular adaptation to Cd toxicity (53). However, prolonged Cd exposure can result in excessive autophagic activity, potentially leading to apoptosis as cells fail to recover from persistent stress (72).

Autophagy and apoptosis are closely linked under metal-induced stress, sharing regulatory pathways such as CHOP-mediated apoptosis, which can be triggered by excessive ER stress (73). In Cd-exposed fish, increased expression of apoptotic markers, including Bax and Casp3, alongside autophagy markers, indicates a transition from adaptive autophagy to programmed cell death (53). The interaction between autophagy and apoptosis under heavy metal stress underscores the delicate balance between cell survival and death. While autophagy initially serves as a protective mechanism, it can eventually lead to apoptosis if stress persists.

##### 3.3.2.2 Autophagy in response to chromium exposure

Chromium (Cr²^+^), the metal of higher toxicity, is globally found in aquatic environments and has been shown to induce disrupt antioxidant defense mechanisms, oxidative stress, and modulate autophagy-related gene expression in fish (*Channa punctatus*) (67).

Exposure to heavy metals, especially Cr²^+^, results in the overproduction of reactive oxygen species (ROS), causing oxidative stress that disrupts cellular homeostasis. In response, autophagy is activated to degrade damaged cellular components and help maintain cell survival (68). In Cr²^+^-exposed *C. punctatus*, genes such as ATG5, LC3, and GABARAP were highly upregulated, indicating enhanced autophagic vesicle creation in response to oxidative stress, while mTOR (Negative regulator of autophagy), was downregulated, suggesting mTOR inhibition-mediated autophagy activation (67).

The accumulation of ROS due to heavy metal exposure can trigger both apoptosis autophagy, with the balance between these processes determining cell fate. Initially, autophagy helps cells adapt to stress, but under prolonged exposure, excessive oxidative damage shifts the response toward apoptosis (74). In Cr²^+^-exposed *C. punctatus*, increased apoptotic markers such as Bax and Caspase-3 were detected alongside autophagy-related genes, indicating a transition from protective autophagy to programmed cell death (67).

##### 3.3.2.3 Copper toxicity and autophagy in crustaceans

Environmental pollutants, particularly metals like copper, induce oxidative stress, leading to the activation of autophagic responses as a protective mechanism. Research has demonstrated that excess copper exposure triggers autophagy in various species, including crustaceans like the Chinese mitten crab (*Eriocheir sinensis*). This activation is often characterized by the upregulation of autophagy-related genes, such as beclin1, LC3, p62, and TFEB (75).

The response of autophagy to copper toxicity is dose dependent. In the case of *E. sinensis*, exposure to lower copper concentrations (0.04–0.18 mg/L) results in the activation of autophagy, marked by the upregulation of atg7 and p62 in the gills (75). However, exposure to higher concentrations (0.7 mg/L) downregulates these markers, suggesting that while low doses of copper trigger protective autophagy, excessive exposure can impair the autophagic process (75). This highlights the complexity of autophagy regulation under environmental stress, with copper exposure eliciting both protective and damaging effects depending on the concentration.

The hepatopancreas of *E. sinensis* appears to be particularly sensitive to copper exposure, demonstrating higher copper accumulation compared to other tissues (76). This organ is essential for detoxification in crustaceans, making it more vulnerable to the effects of copper toxicity. Furthermore, the AMPK pathway has been implicated in the regulation of autophagy in response to copper-induced stress. This signaling pathway helps coordinate the response to oxidative stress by modulating autophagy and related processes (77, 78).

#### 3.3.3 Autophagy in response to nanoparticles

Autophagy plays a crucial role in mitigating the effects of environmental pollutants in aquatic invertebrates, particularly bivalves and other mollusks. Studies on mussels exposed to nanoparticles, such as glass wool from oil spill barriers, have revealed significant lysosomal instability, leading to oxidative stress and lipofuscin accumulation. This autophagic response is essential for managing cell damage and preventing cell death following nanoparticle exposure (79). Similarly, in the bivalve *Ruditapes decussatus*, exposure to cadmium (Cd) has been shown to induce autophagy, which helps maintain cellular function by protecting vital tissues, such as the gills and digestive gland, from toxic damage (80). However, recent advances suggest that autophagy can act as a double-edged sword in response to nanoparticle exposure. For instance, Chen et al. (81) argue that while autophagy may initially serve a cytoprotective function, excessive or dysregulated autophagy induced by certain nanomaterials could exacerbate cellular damage, contributing to pathogenesis and cell death (81). Their call for tiered *in vitro*–*in vivo* testing frameworks highlights the urgency to better characterize the threshold at which autophagy shifts from protective to detrimental, especially given the increasing use of engineered nanoparticles (ENPs) in aquatic environments.

Furthermore, Dube et al. (82) stress that the fate and transformation of ENPs in aquatic systems-through aggregation, dissolution, or interaction with organic matter-can significantly influence their toxicity profiles (82). They point out that autophagy is not only an early cellular response but may also play a role in the systemic effects of ENP exposure, such as bioaccumulation and biomagnification along trophic levels. This raise concerns that chronic ENP exposure might impair autophagic flux in aquatic animals, disrupting tissue function and ecosystem stability. Together, these findings highlight that while autophagy is a critical defense mechanism, it is also a sensitive indicator of nanoparticle-induced stress, and its modulation by different types of NPs must be carefully evaluated to distinguish adaptive responses from toxic outcomes (81, 82).

Studies in fish have also demonstrated contaminant-induced autophagy. For instance, zebrafish (*D. rerio*) exposed to nanoparticles exhibit autophagic responses through the cellular uptake of these particles, highlighting autophagy’s protective role in vertebrate aquatic species as well (83, 84). This stress-induced autophagy contributes to the removal of damaged organelles and maintenance of cellular homeostasis, underscoring a conserved mechanism across aquatic organisms in combating nanoparticle-related environmental stress.

#### 3.3.4 Autophagy in response to hydrocarbons

Beyond nanoparticles, many aquatic invertebrates, including corals and other mollusks, utilize autophagy as a defense mechanism against pollutant-induced stress (85). The blue mussel (*Mytilus edulis*), a widely recognized bioindicator of ecosystem health, exhibits autophagic responses to contaminants. Its digestive gland plays a pivotal role in detoxification, sequestering pollutants into cellular vesicles and lysosomes, thereby activating autophagy to facilitate both food digestion and detoxification (86).

Exposure to lipophilic xenobiotics, such as oil-derived aromatic hydrocarbons (AHs), has also been shown to trigger autophagy, with simulations suggesting that AH exposure and food deprivation serve as key models for understanding autophagic regulation in aquatic organisms (87). Additionally, autophagy is hypothesized to mitigate oxidative stress caused by the accumulation of damaged proteins and lipids in lysosomes, potentially enhancing resilience against pollutant-induced oxidative damage (88).

#### 3.3.5 Hypoxia-induced autophagy

Hypoxia disrupts oxygen homeostasis, leading to excessive reactive oxygen species (ROS) generation and oxidative damage in fish gills (32, 89). This stress activates autophagy, primarily regulated by the hypoxia-inducible factor (HIF)-Bcl-2/adenovirus E1a 19 kDa interacting protein 3 (BNIP3) pathway, which promotes autophagosome formation (90). In *Ctenopharyngodon idella*, exposure to hypoxia significantly upregulated autophagy-related genes, including BNIP3, ATG5, ATG12, and Beclin1, alongside an increase in LC3-II protein levels, indicating enhanced autophagic activity (91).

While autophagy initially acts as a protective mechanism, prolonged hypoxia may lead to excessive autophagic flux, contributing to apoptosis (92). The activation of caspase-dependent pathways and upregulation of apoptotic markers, such as Bax and Caspase-3, indicate a shift from survival to programmed cell death in severely stressed fish (93). The transition from autophagy to apoptosis is closely linked to endoplasmic reticulum stress (ERS), where key regulatory proteins, including CHOP, ATF4, and XBP1s, are upregulated under hypoxia but mitigated by protective compounds such as tea tree oil (93).

In aquatic invertebrates, particularly intertidal sessile species like mussels and oysters, hypoxia is a recurrent environmental stressor due to fluctuating tidal levels. These organisms show remarkable hypoxia tolerance, partly due to adaptive regulation of autophagy, apoptosis, and mitochondrial quality control mechanisms. *Crassostrea gigas* and *Mytilus edulis*, for instance, exhibit distinct responses to hypoxia and subsequent reoxygenation. Long-term hypoxia upregulates Bcl-2 in both species, promoting anti-apoptotic signaling (94). However, *M. edulis* shows stronger transcriptional activation of apoptotic and inflammatory markers (Caspases, BAX, NF-κB), while *C. gigas* demonstrates a muted response, suggesting a greater capacity to maintain cellular homeostasis under oxygen stress (94). Hypoxia/reoxygenation (H/R) stress also affects mitochondrial integrity in these bivalves. In mussels, H/R induces mitochondrial fission, suppression of fusion, and activation of mitophagy-highlighting the role of mitochondrial quality control in hypoxia resilience. Conversely, oysters show stable expression of mitochondrial maintenance genes under similar stress, indicating superior inherent tolerance (95).

Transcriptomic analysis of *Mytilus chilensis* under hypoxia and reoxygenation reveals over 15,000 differentially expressed genes in gill tissue alone, including regulators of Toll-like, mTOR, and apoptosis pathways, signifying immunometabolic reprogramming under low oxygen (96). These changes support a shift from aerobics to anaerobic metabolism and may increase disease susceptibility due to immune suppression. Earlier studies further corroborate the autophagic role in coping with fluctuating oxygen levels. Autophagy induced by anoxia in *M. edulis* and *M. galloprovincialis* was shown to be reversible and protective, facilitating lysosomal stability and the clearance of ROS-damaged components (97). This basal level of autophagic activity may underlie the resilience of intertidal invertebrates to repeated hypoxic episodes. Together, these findings underscore that hypoxia-induced autophagy is a conserved yet species-specific response across aquatic organisms. While it serves as a survival strategy in both fish and invertebrates, the molecular pathways involved and the balance between autophagy, apoptosis, and mitochondrial maintenance vary significantly, reflecting differences in ecological adaptation and physiological tolerance (32, 93).

#### 3.3.6 Autophagy and pesticide exposure

Various environmental pollutants, including pesticides such as fipronil, can trigger autophagy in aquatic organisms, often as a protective response to stress-induced cellular damage (56). Environmental stressors such as pesticide exposure, oxidative stress, and nutrient depletion can induce autophagy by activating key regulatory pathways. Fipronil exposure in *Cyprinus carpio* led to significant upregulation of autophagy-related genes (ATG5, ATG12, ATG16L, LC3-II, and Beclin1), suggesting enhanced autophagic activity as an adaptive response (56). The ubiquitination pathway (ATG5–ATG12–ATG16L) has been reported to be crucial for autophagosome formation and degradation (98) Similarly, increased expression of Beclin1, a key autophagy regulator, further confirms autophagy activation following environmental pollutant exposure (99, 100).

While autophagy is a survival mechanism under stress, prolonged exposure to pollutants can lead to excessive autophagic flux, pushing cells toward apoptosis (101). In fipronil-exposed carp, autophagic activity was particularly evident in liver and intestinal tissues during the early exposure period, as indicated by increased LC3-II/LC3-I ratios. However, prolonged exposure resulted in a shift toward apoptosis, with increased expression of apoptotic markers such as Bcl-2 and Caspase-3 (56). The balance between autophagy and apoptosis is critical in determining cell fate under environmental stress.

## 4 Autophagy monitoring methods

Monitoring autophagy in aquatic animals is essential for understanding their roles in development, immunity, disease progression, and responses to environmental stress. A variety of methodological approaches have been developed and adapted across numerous aquatic species, each offering specific strengths and limitations. Commonly used techniques include Western blotting for detecting autophagy-related protein markers (e.g., LC3, p62), qRT-PCR for analyzing the transcriptional regulation of autophagy-related genes, and fluorescence microscopy for visualizing autophagic structures in live or fixed cells. Additional tools such as flow cytometry, transmission electron microscopy (TEM), and immunohistochemistry further enhance the ability to quantify and localize autophagic components within tissues (102) (**Table 2**). When applied in combination, these methods provide a comprehensive understanding of autophagy dynamics and its physiological significance in aquatic organisms (112, 113). Building upon this methodological foundation, autophagy has emerged as a critical biomolecular marker and therapeutic target in aquaculture. Reflecting their importance on mammals, modulating autophagy in aquatic species offers valuable insights into disease mechanisms and control strategies (6). A variety of pharmacological agents have been utilized to explore and manipulate this pathway. For example, chloroquine (CQ), a widely used autophagy inhibitor, effectively blocks the fusion of autophagosomes with lysosomes, thereby halting autophagic flux in both *in vitro* and *in vivo* fish models (114–117). On the other hand, Autophagy inducers such as rapamycin- an inhibitor of the mTOR signaling pathway have been shown to enhance autophagic activity by modulating key autophagy-related genes and proteins, as demonstrated in *Litopenaeus vannamei*. This induction of autophagy contributes to improved cellular homeostasis and may strengthen immune responses during pathogenic challenges (116). These modulators not only serve as experimental tools but also reveal the profound role of autophagy in host-pathogen dynamics, contributing to our understanding of fish immunity and stress resilience. Importantly, leveraging these compounds in aquaculture holds transformative potential: they may pave the way for new interventions to increase disease resistance, optimize vaccine responses, and cultivate genetically or physiologically robust aquatic species populations, advancing sustainability in aquatic food production.

**Table 2:** Methods for monitoring autophagy.

Method	Description	Advantages	Limitations	References
Western Blotting	Detects protein markers of autophagy (e.g., LC3B-II, SQSTM1/P62, ATG proteins).	Quantitative, widely used, detects protein-level changes.	Requires careful controls (e.g., lysosomal inhibitors), can be challenging to interpret.	(103, 104)
PCR-Based Assays	Measures mRNA levels of autophagy-related genes (e.g., atg genes).	Sensitive, useful for gene expression studies.	mRNA levels do not directly reflect autophagic activity due to post-translational regulation.	(105, 106)
Transmission Electron Microscopy (TEM)	Directly visualizes autophagosomes and autolysosomes at an ultrastructural level.	High-resolution, gold standard for autophagy detection.	Labor-intensive requires specialized equipment and expertise.	(103, 107)
Flow Cytometry	Measures autophagy in single cells using fluorescent dyes like Cyto-ID^®^, which label autophagic vacuoles. Used to analyze large numbers of hemocytes in aquatic species.	Fast, sensitive, can measure both autophagy and cell health at the same time; good for tracking changes over time.	Needs specialized equipment and careful setup for accurate results; may need adjustments for different species.	(108, 109)
Transmission Electron Microscopy (TEM)	Uses high-resolution imaging to directly see tiny cell structures like autophagosomes and autolysosomes, which are involved in autophagy.	Gives clear, detailed images of autophagic structures; considered the gold standard for confirming autophagy at the cellular level.	Time-consuming, needs special equipment and expert skills to prepare and analyze samples.	(109)
Immunohistochemistry (IHC)	Uses antibodies to detect and locate autophagy-related proteins (like LC3, SQSTM1) in tissue slices, helping show where autophagy is happening in different parts of a tissue.	Shows both the presence and location of proteins involved in autophagy; useful for understanding tissue-specific responses.	Needs well-prepared tissue samples and specific antibodies; interpretation can be affected by tissue quality and staining conditions.	(35, 110, 111)

### 4.1 Western blotting

Western blotting is a widely used technique for monitoring autophagy by detecting specific protein markers associated with the autophagic process. In aquatic animals, it is commonly employed to identify the conversion of LC3-I to LC3-II, a hallmark of autophagosome formation, as well as to quantify other autophagy-related proteins such as SQSTM1/p62, BECLIN 1, ATG5, and ATG7 (102, 118). However, these markers are not fully specific to autophagy, and care must be taken when interpreting the data (119). A key challenge is distinguishing between increased autophagy and impaired lysosomal degradation; thus, lysosomal inhibitors such as chloroquine, bafilomycin A1, or 3-MA are often used to assess autophagic flux (108). Experimental accuracy depends on several factors, including antibody specificity, membrane type, and exposure duration. Comparing LC3-II levels to a stable housekeeping protein is generally more reliable than using LC3-I as a reference (102). Despite its limitations, when paired with appropriate controls and complementary assays, Western blotting remains a valuable and reproducible method for studying autophagy in aquatic species (103).

In aquatic species, Western blotting has been successfully applied to assess autophagic responses under various physiological and pathological conditions. For instance, in oysters (*Crassostrea gigas*), the technique has been used to detect autophagy-related proteins post-infection with OsHV-1 and *Vibrio aestuarianus*, indicating the activation of innate immune mechanisms (119). Yang et al. (118) employed Western blotting to examine hemocyte protein expression using SDS-PAGE, specific primary antibodies (e.g., anti-CgLC3), and chemiluminescence-based detection systems (118). Similarly, Zhang et al. (102) demonstrated oxidative stress-induced autophagy by visualizing LC3 levels in oyster hemocytes (102). Picot et al. (108) further characterized the expression of autophagy-related genes such as MAP1LC3, BECN1, MTOR, and SQSTM1 across different tissues of *C. gigas*, confirming a conserved and functional autophagy pathway (108). Collectively, these examples highlight Western blotting’s pivotal role in elucidating the molecular regulation of autophagy in diverse aquatic organisms (104, 120).

### 4.2 PCR-based assays

PCR-based assays, particularly real-time PCR, are widely employed to explore the transcriptional regulation of autophagy-related genes (atg genes) in aquatic organisms. These assays provide valuable insights into gene expression levels, helping to identify changes in autophagy-related pathways under various conditions. However, it is important to note that mRNA expression does not always directly correlate with autophagic activity due to complex posttranslational regulation (105, 106). Despite this limitation, PCR remains a widely used method for studying autophagy, especially when combined with other techniques. It has been used to investigate factors such as circadian rhythms in atg gene expression, the influence of bacterial infections on host autophagy, and the role of specific genes in immune responses (121, 122). PCR can also be instrumental in examining the timing of gene expression during different developmental stages, as well as in response to environmental or physiological stressors.

For example, PCR analyses have demonstrated that several atg genes are maternally deposited in zebrafish embryos, with distinct expression patterns observed during early development. Some of these genes exhibit stable expression, while others show dynamic changes, indicating their potential roles in regulating embryonic development (122). In this way, PCR-based assays offer critical insights into the temporal regulation of autophagy genes. Although PCR cannot provide direct functional evidence of autophagic flux, it is an essential complementary tool when combined with protein-level analyses and functional assays. This integration enhances the overall understanding of autophagy regulation at both the transcriptional and functional levels (89).

### 4.3 Fluorescence microscopy

Fluorescence microscopy is a powerful technique for visualizing and quantifying autophagy at the cellular level. In aquatic species, it enables spatial resolution of autophagic structures using fluorescent dyes like Cyto-ID^®^, which selectively stain autophagic vacuoles, allowing direct image-based analysis of fluorescence intensity in individual cells (109). This technique is particularly advantageous for live-cell imaging and is often used in conjunction with confocal microscopy for enhanced resolution. In zebrafish, fluorescence microscopy is especially effective due to the natural transparency of embryos and larvae, permitting real-time observation of autophagic processes (102). Transgenic models such as Tg (CMV: GFP-map1lc3) express fluorescently tagged LC3B to mark autophagosomes as distinct puncta (102, 118). Advanced dual fluorescence reporters like mCherry-GFP-LC3B further improve accuracy by distinguishing between autophagosomes and autolysosomes based on pH-sensitive signal degradation (109). Additionally, specialized transgenic lines targeting organelles (e.g., mitochondria) or pathogens with fluorescent tags enable the study of selective autophagy, including mitophagy and xenophagy (118).

This method has been effectively applied to a range of aquatic organisms. In the Pacific oyster (*Crassostrea gigas*), Picot et al. (109) demonstrated that adapted mammalian protocols using Cyto-ID^®^ and immunofluorescence microscopy could visualize autophagic activity in hemocytes (109). Zhang et al. (102) used this approach to show that lipopolysaccharide (LPS)-induced oxidative stress significantly increased autophagosome formation, with fluorescent staining revealing the accumulation of autophagic vacuoles (102). The application of fluorescence microscopy in both vertebrates and invertebrates highlights its versatility and effectiveness in tracking autophagy-related responses under immune challenge, developmental stages, and environmental stress conditions (123).

### 4.4 Flow cytometry

Flow cytometry is a high-throughput technique used to quantify autophagic activity at the single-cell level by measuring fluorescence intensity of specific dyes, such as Cyto-ID^®^, that selectively label autophagic vacuoles. In aquatic species, this method enables rapid and accurate detection of autophagy in large hemocyte populations, making it a valuable tool for immune and stress response studies (109). When combined with viability stains (e.g., 7-AAD or PI), flow cytometry can simultaneously assess cell viability and autophagic status, offering a comprehensive understanding of host responses under various experimental conditions (108). The technique’s sensitivity and capacity for multiparametric analysis make it especially suitable for evaluating dynamic changes in autophagy during pathogen infection or environmental stress in invertebrates and fish species.

Flow cytometry has been successfully applied in the Pacific oyster (*Crassostrea gigas*) to monitor autophagy in hemocytes under viral stress. Picot et al. (109) adapted mammalian protocols for invertebrate application, using the Cyto-ID^®^ Autophagy Detection Kit to label autophagic compartments (109). Later, Picot et al. (108) employed flow cytometry to quantify hemocytes containing autophagosomes during OsHV-1 infection, analyzing approximately 5000 gated events per sample while excluding debris and bacteria through size discrimination (108). This approach revealed temporal and tissue-specific modulation of autophagic activity, indicating the involvement of autophagy in antiviral immune responses. These findings support the utility of flow cytometry as a quantitative and scalable method to study autophagy in aquatic immunology research.

### 4.5 Transmission electron microscopy

Transmission electron microscopy (TEM) is a high-resolution imaging technique used to observe the ultrastructural features of cells, providing detailed views of cellular processes, including autophagy. TEM is particularly valuable for visualizing autophagic vesicles, such as autophagosomes and autolysosomes, which appear as double- or single-membrane-bound structures within the cytoplasm. This technique is critical for confirming the presence of autophagic structures and understanding the morphological details of autophagic processes at the subcellular level. TEM has become an essential tool for assessing immune responses in aquatic species, as it allows for the direct observation of cellular degradation pathways involved in pathogen clearance and stress adaptation (109).

In aquatic species, TEM has been successfully applied to examine autophagic activity in hemocytes. For instance, Picot et al. (109) used TEM to investigate autophagy in hemocytes of the Pacific oyster (*Crassostrea gigas*) (109). Their study revealed autophagic vesicles within the hemocytes, confirming that the autophagic process is activated during immune responses, especially in reaction to pathogen exposure. TEM provided clear visual evidence of autophagosome formation and their subsequent degradation, offering valuable insights into the cellular mechanisms employed by oysters in defending against infections.

### 4.6 Immunohistochemistry

Immunohistochemistry (IHC) is a widely used technique to detect and localize autophagy-related proteins in tissue sections, providing spatial context to cellular responses. The method involves fixation of tissues, typically in formalin, followed by paraffin embedding, sectioning, and staining. Specific primary antibodies targeting autophagy markers such as LC3, Beclin-1, SQSTM1/p62, or mTOR are used to label the proteins of interest, and these are subsequently detected using enzyme-conjugated secondary antibodies (35). The antigen–antibody complexes are visualized with chromogenic substrates like DAB (3,3′-diaminobenzidine), producing a brown precipitate at the site of expression, often counterstained with hematoxylin to distinguish cellular structures. IHC is especially useful for determining the localization of autophagy activity within tissue architecture, making it an important complement to molecular techniques such as Western blotting or qRT-PCR.

This technique has been effectively applied in aquatic invertebrates, notably in the Pacific oyster (*Crassostrea gigas*), to investigate immune responses against pathogens. Picot et al. (35) used IHC to localize autophagy-related markers LC3 and SQSTM1 in oyster hemocytes and digestive glands during OsHV-1 infection (35). Their study revealed differential expression patterns in various tissues, suggesting activation of the autophagy pathway in response to viral challenge. IHC allowed the researchers to visualize not only the presence but also the distribution and intensity of autophagy-related proteins, providing critical insights into tissue-specific autophagic responses in infected oysters (110, 111).

## 5 Autophagy in host-pathogen interactions

Autophagy is crucial for preserving cellular homeostasis and regulating cell health, particularly in response to infections (48, 124). Although autophagy has been extensively studied in mammals, its investigation in aquatic species remains relatively underexplored (125, 126). This is partly due to the limited availability of suitable detection methods, validated molecular markers, specific antibodies, and autophagy modulators in aquatic research compared to mammalian systems (6). Despite these constraints, emerging studies suggest that autophagy in aquatic species plays a vital role in various biological processes, including resistance to toxins and pathogens (125).

**Table 3** summarizes the diverse roles of autophagy in aquatic animals, encompassing embryonic development, metabolism, and host-pathogen interactions. During embryogenesis, autophagy is essential for processes such as neurogenesis, tissue morphogenesis, and angiogenesis. Studies in zebrafish embryos have demonstrated that genes like atg5 and wdr24 are crucial for proper brain and organ development, with their disruption leading to morphological defects and increased cell death (120, 127, 129, 131–133). In metabolic regulation, autophagy contributes to protein turnover, lipid homeostasis, and compensatory growth. Dietary supplementation with selenium and zinc modulates autophagic flux to enhance flesh quality and reduce hepatic lipid accumulation, while early-life nutrition and miRNA regulation also influence autophagy-linked metabolic pathways in several fish species (8, 114, 136–138). Autophagy also plays a dual role in host-pathogen interactions. On one hand, it enhances immune defenses by limiting viral and bacterial replication, as observed in responses against VHSV, *Aeromonas hydrophila*, and other pathogens (115, 143, 144). On the other hand, certain viruses such as SVCV, ISAV, and ISKNV manipulate autophagy to support their replication and persistence within host cells (23, 33, 139, 140, 146).

**Table 3:** Autophagy in pathogen defense, metabolism, and embryonic development.

Study Focus/Process	Cell Model/Organism	Key Findings	References
Embryonic development			
Autophagy in embryonic development	Zebrafish embryos	High autophagy activity observed, upregulated by rapamycin and calpeptin; GFP-tagged Lc3 and Gabarap used to track autophagic activity	(127)
Liver-specific autophagy in embryonic development	Transgenic zebrafish larvae	EGFP-Lc3 puncta increased with autophagy inducer Torin1; further accumulation with chloroquine due to blocked lysosomal degradation	(128)
Embryonic morphogenesis	Transgenic zebrafish embryos	Autophagy active in multiple tissues, including the heart; inhibition caused morphogenesis defects, increased cell death, abnormal heart structure, and reduced survival	(129)
Early embryonic development	Zebrafish embryos	Maternal factors drive early development before zygotic genome activation, influencing axis formation, germ layer specification, and other early embryonic processes.	(130)
Role of atg5 in neurogenesis and organogenesis	Zebrafish embryos	atg5 is essential for brain development, body plan formation, and regulation of neural gene expression.	(106)
Autophagy in angiogenesis and vascular remodeling	Zebrafish embryos	CPCD inhibits angiogenesis by blocking VEGFR2/AKT signaling and induces autophagy, reducing blood vessel sprouting.	(131)
Autolysosome formation and embryonic senescence	Zebrafish embryos	Spns1 and v-ATPase cooperate in autolysosomal biogenesis and acidification. Loss of Spns1 leads to embryonic senescence, which can be suppressed by disrupting v-ATPase.	(132)
Embryonic development	Zebrafish embryos	Wdr24 is required for normal embryogenesis; knockdown causes developmental defects and cell death due to dysregulated autophagy.	(133)
GABARAP expression during embryonic development	*Haliotis diversicolor* (small abalone)	GABARAP was expressed throughout embryonic and larval stages, with peak levels at the gastrula stage.	(134)
Temperature-induced autophagy and apoptosis during embryogenesis	*Sepiella japonica* embryos	High temperature altered autophagy gene expression (LC3, BECN1, Inx4) and increased embryonic sensitivity, highlighting autophagy’s role in temperature-regulated development.	(135)
Metabolism			
Protein degradation and flesh quality	Rainbow trout (*Oncorhynchus mykiss*)	Supranutritional dietary selenium inhibited autophagy-related protein degradation, improving fillet firmness and water-holding	(136)
Compensatory growth and protein metabolism	Arctic charr (*Salvelinus alpinus*)	Autophagy-related genes regulate compensatory growth; miRNAs influence glycogen homeostasis.	(137)
Zinc and hepatic lipid metabolism	Hepatocytes (liver cells)	Zinc reduces hepatic lipid accumulation via Zn2+/MTF-1/PPARα and Ca2+/CaMKKβ/AMPK pathways, promoting lipophagy.	(138)
Compensatory growth and protein metabolism	Arctic charr (*Salvelinus alpinus*)	Autophagy-linked protein accretion, miRNA-regulated glycogen homeostasis	(137)
Fish metabolism regulation	Various fish species	Autophagy, miRNAs, genome complexity, and early-life nutrition impact metabolism	(8)
Starvation-induced autophagy and metabolism	*Mytilus* spp. (mussel)	Starvation upregulated autophagy genes (atg2, atg6, atg13) and activated metabolic and lysosomal pathways in Mytilus.	(119)
Pathogen Infection			
Spring Viraemia of Carp Virus (SVCV)	Epithelioma papulosum dellad (EPC) cells	SVCV glycoprotein activates autophagy, supporting viral replication and cell survival.	(33)
Rhabdoviral G Glycoproteins Induce Autophagy	Zebrafish, Vertebrate Cell Lines	Rhabdoviral G glycoproteins (VSV, VHSV, SVCV) induce autophagy, suggesting its role in antiviral defense.	(139)
Autophagy Induction by Infectious Salmon Anemia Virus (ISAV)	Atlantic Salmon Cells	ISAV induces autophagy, shown by LC3-GFP redistribution and autophagosome formation, suggesting a role in viral replication.	(140)
Autophagy in Infectious Spleen and Kidney Necrosis Virus (ISKNV) Replication	CPB (Chinese perch brain) Cells	ISKNV triggers autophagy, which promotes viral replication but reduces extracellular virus yields.	(23)
Autophagy in TDCIPP-Induced Neurotoxicity	Zebrafish embryos and larvae	Exposure to TDCIPP triggers autophagy, which helps reduce developmental neurotoxicity in zebrafish	(141)
Autophagy in nucleated erythrocytes and antiviral immunity	Turbot (*Scophthalmus maximus*) erythrocytes	Autophagy in red blood cells (RBCs) limits the replication of viral hemorrhagic septicemia virus (VHSV) and boosts antiviral immunity.	(115)
ROS-induced autophagy in antiviral defense	Ctenopharyngodon della kidney cells	ROS-induced autophagy restricts grass carp reovirus (GCRV) replication. HSP70 and HMGB1b promote autophagy by interacting with Beclin 1.	(142)
γ-Aminobutyric Acid (GABA) Signaling in Antibacterial Autophagy	Macrophages, mice, zebrafish	Activation of γ-Aminobutyric acid (GABA) enhances autophagy, facilitates phagosomal maturation, and strengthens antimicrobial defense against intracellular bacterial infections.	(143)
Autophagy in immune response to bacterial infection	*Macrobrachium rosenbergii* (freshwater prawn)	Autophagy reduces apoptosis and improves survival during Aeromonas hydrophila infection.	(144)
Autophagy in OsHV-1 and *Vibrio aestuarianus* infections	*Crassostrea gigas* (Pacific oyster)	Autophagy is active and protective against OsHV-1 and *V. aestuarianus*; its induction improves survival, while inhibition increases mortality.	(119)
Autophagy in response to *Vibrio tapetis* infection	*Mytilus galloprovincialis* hemocytes	*V. tapetis* induces autophagosome formation and LC3-II expression; autophagy appears protective and is blocked by Wortmannin	(145)
Autophagic and lysosomal responses to environmental stress	*Mytilus edulis* and *Mytilus galloprovincialis* hepatopancreas cells	Environmental stressors (e.g., PAHs, copper, starvation) trigger lysosomal damage and autophagy; starvation-induced autophagy protects against oxidative stress.	(97)

Beyond its involvement in individual infections, autophagy is fundamentally intertwined with the development and function of the immune system. It supports both innate and adaptive responses and contributes directly to the clearance of intracellular pathogens (33). However, many pathogens have evolved mechanisms to evade or exploit autophagic pathways to their advantage (147), complicating their overall role. This complexity suggests that autophagy’s activity during infection is highly context-dependent, influenced by specific pathogen-host interactions. A deeper understanding of these dynamics is essential for improving aquatic immunology and developing targeted disease management strategies in aquaculture (148).

### 5.1 Autophagy in viral infections

Extensive research has explored the importance of autophagy in viral infections in mammals; however, studies on viral infections in fish remain relatively limited (149, 150). The first document autophagy of virus-induced in fish cells came from Schiøtz et al. (140), who demonstrated that Infectious Salmon Anemia Virus (ISAV) induces autophagy in Atlantic salmon cells (140). This was evidenced by the formation of autophagosomes and the redistribution of LC3-GFP fluorescence (140). Inhibiting autophagosome formation led to a reduction in LC3-GFP puncta and viral production, suggesting that autophagy facilitates ISAV replication (140). Similarly, Liu et al. (33) showed that Spring Viremia of Carp Virus (SVCV) activates autophagy in infected cells, promoting viral replication and survival (33). This process, triggered by the SVCV glycoprotein, is mediated through the ERK/mTOR pathway, with autophagy suppression significantly reducing SVCV replication.

Building on these individual findings, recent reviews provide broader insight into the dual and context-specific roles of autophagy in aquatic viral infections. Li et al. (10) reported that both RNA and DNA viruses manipulate host autophagy-either enhancing their replication or being suppressed by it. For example, autophagy supports replication in SVCV and ISAV infections but acts protectively against IHNV and OsHV-1 (10). Key pathways like PI3K/AKT/mTOR and eIF2α are central to this modulation (10). These insights suggest that targeting autophagy signaling could offer strategies for antiviral therapy and vaccine enhancement in aquaculture.

Transcriptomic studies indicate that rhabdoviral G glycoproteins from both mammalian and fish viruses induce autophagy in vertebrate cells, contributing to antiviral immunity. Rhabdoviruses encode a single surface glycoprotein (G protein), which is essential for viral entry by mediating receptor binding and membrane fusion. Beyond its structural role, the G protein also influences host cellular responses, including autophagy. For instance, Luo et al. (151) identified the laminin receptor (LamR) as a functional receptor for Micropterus salmoides rhabdovirus (MSRV), demonstrating that the G protein directly binds to LamR to facilitate viral attachment and internalization via clathrin-mediated endocytosis (151). These findings underscore the G protein’s dual role in mediating virus entry and potentially modulating intracellular pathways, including autophagy (151). Given the established links between endocytic trafficking and autophagy, such interactions may be central to understanding how rhabdoviruses exploit host cellular machinery. Furthermore, peptides in the fusion domains of rhabdoviral G proteins have been shown to trigger autophagy, suggesting that modulating this process could be a promising strategy for preventing and treating rhabdoviral infections such as rabies (139). Infectious Spleen and Kidney Necrosis Virus (ISKNV), a severe pathogen affecting fish, particularly Chinese perch (*Siniperca chuatsi*), also activates autophagy in infected cells (23). While autophagy promotes viral replication, it simultaneously restricts viral release. Notably, blocking autophagy results in the release of a higher number of infectious viral particles (139).

Further studies highlight the role of metabolic stress in antiviral defense via autophagy. Sun et al. (146) demonstrated that glutamine starvation induces autophagy, which inhibits Snakehead Fish Vesiculovirus (SHVV) replication in snakehead fish (*Channa striata*) (146). Similarly, Zhao et al. (152) found that autophagy plays a protective role during Infectious Hematopoietic Necrosis Virus (IHNV) infection. Inducing autophagy suppressed viral replication, whereas blocking it enhanced infection (152). IHNV, which primarily affects salmonid fish, triggers autophagy, leading to autophagosome formation and altered LC3 protein levels. Enhancing autophagy reduces viral replication, whereas inhibiting it results in an increased viral load, suggesting that autophagy functions as a defense mechanism against IHNV (152).

Additionally, teleost fish red blood cells (RBCs) contribute to antiviral defense through autophagy. Pereiro et al. (115) found that Nk-lysin (Nkl), an antimicrobial peptide associated with viral resistance, is localized inside autophagosomes, indicating a link between autophagy and antiviral immunity (115). Their study demonstrated that autophagy in RBCs enhances antiviral defense, as blocking autophagy led to increased viral replication. This discovery provides the first evidence that autophagy in RBCs plays a crucial role in antiviral immunity in teleost fish.

Overall, these findings underscore the dual role of autophagy in viral infections. While autophagy can support viral replication in some cases, it also serves as a key antiviral defense mechanism in others. Recent studies on Grass Carp Reovirus (GCRV) further emphasize the dual role of autophagy during viral infections in fish. Chu et al. (153) demonstrated that GCRV infection stimulates autophagy in grass carp spleen and CIK cells, where enhanced autophagy-either induced by rapamycin or CiATG5 overexpression-suppressed viral replication and mitigated excessive inflammatory responses (153). Their findings suggest that autophagy serves as a protective mechanism by promoting viral clearance and attenuating cytokine-mediated damage. In contrast, Zhu et al. (154) found that GCRV-induced autophagy in CIK cells facilitates viral replication via the Akt-mTOR signaling pathway. In this study, activation of autophagy enhanced viral proliferation, while its inhibition reduced viral titters, indicating that autophagy supports the virus lifecycle under certain conditions (154). Together, these contrasting findings illustrate the complex, context-dependent nature of autophagy in virus-host interactions, highlighting the need for deeper investigation into the underlying molecular pathways. A better understanding of these mechanisms in fish could contribute to the development of novel strategies for controlling viral infections in aquaculture.

Autophagy also plays a protective role against viral infections in invertebrates such as the Pacific oyster (*Crassostrea gigas*), particularly during Ostreid herpesvirus 1 (OsHV-1) outbreaks (119). Autophagy is functionally active in oysters, with conserved autophagy-related genes (e.g., ATG1, BECN1) and the autophagosome marker LC3-II being upregulated upon infection (119). Induction of autophagy through starvation or carbamazepine significantly enhanced oyster survival and reduced viral DNA levels (119, 155). Although NH_4_Cl inhibited autophagy in mantle and hemolymph tissues, this inhibition was not directly associated with increased viral replication, suggesting that the relationship between autophagy and OsHV-1 may be tissue-specific and more complex than a straightforward antiviral mechanism (108). Moreover, oyster families with low susceptibility to OsHV-1 showed early upregulation of autophagy genes, suggesting that effective autophagy activation is linked to viral resistance, highlighting its role as a vital innate immune mechanism in mollusks (119).

### 5.2 Autophagy in bacterial infections

Recent studies underscore the multifaceted role of autophagy in bacterial infections in aquatic species, functioning as both a protective and, in some contexts, a detrimental mechanism (6). As part of the innate immune system, autophagy is rapidly activated upon pathogen invasion in aquatic species, like higher vertebrates (156–159). Its antibacterial functions have been well demonstrated in species like Japanese flounder, where *Edwardsiella tarda* infection triggers autophagic degradation through the pol-miR-3p-2–p53–Beclin-1 pathway (159–162). Comparable protective responses are observed in zebrafish infected with Shigella flexneri, *Mycobacterium marinum*, and *Salmonella typhimurium*, where the silencing of autophagy-related genes such as atg5, p62, and Dram1 exacerbates infection severity (160–162). However, emerging evidence also highlights the role of autophagy in bacterial immune evasion. For instance, in *Miichthys miiuy*, infection with *Vibrio harveyi* induces eIF3k expression, promoting autophagy-mediated degradation of MyD88, thereby hindering NF-κB signaling and inflammatory response activation (156). Similarly, in *Staphylococcus aureus*-infected zebrafish, LC3-associated phagosomes may serve as replication sites, facilitating bacterial escape into the cytoplasm (163, 164). Furthermore, autophagy has been implicated in ferroptosis-induced death of host cells, as seen in *E. coli*-infected grass carp red blood cells (GcRBCs), where autophagy-mediated iron release initiates oxidative damage and cell death (157, 165). These findings collectively suggest that while autophagy is a critical antibacterial mechanism, its regulation and context-dependent outcomes must be carefully considered in aquaculture disease management strategies (152).

In the Hong Kong oyster (*Crassostrea hongkongensis*), autophagy serves as a crucial innate immune response against Vibrio parahaemolyticus infection. Upon bacterial challenge, autophagy was rapidly activated in hemocytes, peaking at 6 hours post-infection-which enhanced bacterial clearance and reduced apoptosis (166). This process was shown to be dependent on AMP-activated protein kinase (AMPK) signaling, regulated by infection-induced surges in AMP and reactive oxygen species (ROS) (166). These findings underscore the essential role of autophagy in host defense and highlight AMP and ROS as upstream co-regulators of autophagic activation during bacterial infection. Similarly, in *Crassostrea gigas* (Pacific oyster), autophagy also plays a protective role against *Vibrio aestuarianus*, a pathogen linked to mortality outbreaks in aquaculture. Experimental infection revealed that inhibition of autophagy with NH_4_Cl reduced LC3-II levels, increased bacterial DNA loads, and heightened oyster mortality, suggesting that suppressed autophagy worsens infection outcomes (119). In contrast, autophagy stimulation with carbamazepine reduced bacterial burden and improved survival, regardless of host susceptibility levels. These findings collectively reinforce the conserved and protective nature of autophagy in bacterial defense across oyster species, albeit through distinct upstream regulatory cues (167, 168).

## 6 Potential biotechnological applications

### 6.1 Probiotics as autophagy modulators in aquaculture

In recent years, the modulation of autophagy has emerged as a promising biotechnological tool in aquaculture, particularly using probiotics (169). Probiotics, which are live, viable microorganisms that provide health benefits (such as strengthening the immune system, enhancing resistance to infectious diseases, and improving tolerance to stressful conditions) to the host when consumed through the diet (170), have been found to regulate autophagy in different cell lines and tissues (171, 172), offering potential applications for improving aquatic animal health and productivity.

Probiotics are increasingly recognized for their ability to modulate autophagy, contributing to the health and survival of aquatic organisms. For instance, studies have demonstrated that *Lactobacillus rhamnosus* can influence physiological processes in zebrafish embryos by regulating both autophagy and apoptosis, ultimately improving developmental outcomes (121). This suggests that probiotics could be utilized to enhance embryonic development and survival rates in aquaculture species.

Beyond developmental benefits, probiotics also enhance disease resistance by modulating autophagy. For example, *Bacillus amyloliquefaciens* SC06 has been reported to induce autophagy in murine macrophage cell lines, providing antibacterial activity against *Escherichia coli* (169). This finding indicates that probiotics could be employed to strengthen immune responses in aquatic species, reducing bacterial infections in aquaculture settings.

The regulatory effects of probiotics on autophagy extend beyond immunity and gut health to include organ protection, stress resistance, and reproductive health. These diverse functions, summarized in **Table 4**, highlight the potential of probiotics as an innovative strategy for improving aquaculture sustainability and productivity.

**Table 4:** Summarizing the functions of probiotics as autophagy modulators.

Function	Probiotic Strain	Mechanism/Effect	Species Studied	Citation
Immune Regulation	*Lactobacillus brevis* BGZLS10-17	Modulates autophagy in mesenteric lymph node cells (MLNC) via ATG5-dependent pathways, enhancing immune responses.	*In vitro* (mouse cells)	(173)
Anti-Inflammatory Effects	*Lactobacillus salivarius* AR809	Reduces inflammatory mediators and elevates autophagic proteins, protecting against S. aureus-induced pharyngitis via Akt-mediated NF-κB and autophagy signaling pathways.	Mice	(174)
Stress Resistance	*Bacillus amyloliquefaciens* SC06	Alleviates oxidative stress-induced intestinal injury by triggering autophagy via p38-mediated pathways.	Rats	(169)
Disease Prevention	*Lactobacillus rhamnosus* GG	Reduces autophagy marker expression and LC3 activity during viral gastroenteritis, preventing tissue damage and maintaining gut homeostasis.	Piglets	(175)
Organ Protection	*Lactobacillus reuteri* ZJ617	Ameliorates LPS-induced liver injury in mice by suppressing autophagy, protecting liver tissue.	Mice	(176)
Gut Health and Homeostasis	Bifidobacterium breve	Induces autophagy in intestinal epithelial cells, promoting survival during stress and protecting against pathogen-induced damage.	Mice	(177)
Growth Performance	*Bacillus amyloliquefaciens* SC06	Improves growth performance and intestinal health in weaned piglets by inducing autophagy, suggesting similar applications in aquaculture.	Piglets	(178)
Embryonic Development	*Lactobacillus rhamnosus*	Modulates autophagy and apoptosis in zebrafish embryo development, improving survival and developmental processes.	Zebrafish	(121)
Cardiovascular Protection	Multi-strain probiotics	Reduces autophagy pathway proteins, attenuating cardiomyocyte fibrosis in obese rats, suggesting potential cardiovascular protection in aquatic species.	Rats	(179)
Neuroprotection	SLAB51 (multi-strain probiotic)	Restores impaired neuronal proteolytic pathways (autophagy), reducing brain damage and cognitive decline in Alzheimeric mice, indicating potential neuroprotective effects in aquatic species.	Mice (Alzheimer’s model)	(180)
Renal Protection	Short-chain fatty acids (SCFAs) from microbiota	Increases autophagy and tubular proliferating cells, preventing acute kidney injury (AKI) induced by ischemia-reperfusion, suggesting potential renal protection in aquatic animals.	Mice	(181)
Anti-Tumor Effects	*Lactobacillus* and *Bifidobacterium* strains	Induces autophagic cell death in tumor cells, suggesting potential applications in controlling tumor growth in aquatic species.	*In vitro* (human cells)	(182)
Pharyngeal Tissue Protection	*Lactobacillus salivarius* AR809	Prevents S. aureus-induced pharyngeal inflammation by regulating mTOR signaling pathway-related autophagy, suggesting applications in protecting aquatic species from inflammatory diseases.	Mice	(183)
Reproductive Health	*Lactobacillus rhamnosus* IMC 501	Regulates ovary physiology in zebrafish by inhibiting follicular apoptosis and improving follicular survival through autophagy modulation.	Zebrafish	(184)

#### 6.1.1 Mechanisms of probiotic-induced autophagy

The ability of probiotics to induce autophagy in aquatic animals involves complex and multifaceted mechanisms (185). Certain probiotic species, such as Lactobacillus and Bacillus, activate autophagy through key signaling pathways, including the mTOR pathway and the upregulation of autophagy-related genes (ATG), such as Atg5, Atg7, and Beclin1 (121). These pathways are essential for degrading damaged cellular components and eliminating intracellular pathogens, thereby promoting cell survival and maintaining tissue integrity.

**Figure 3:**
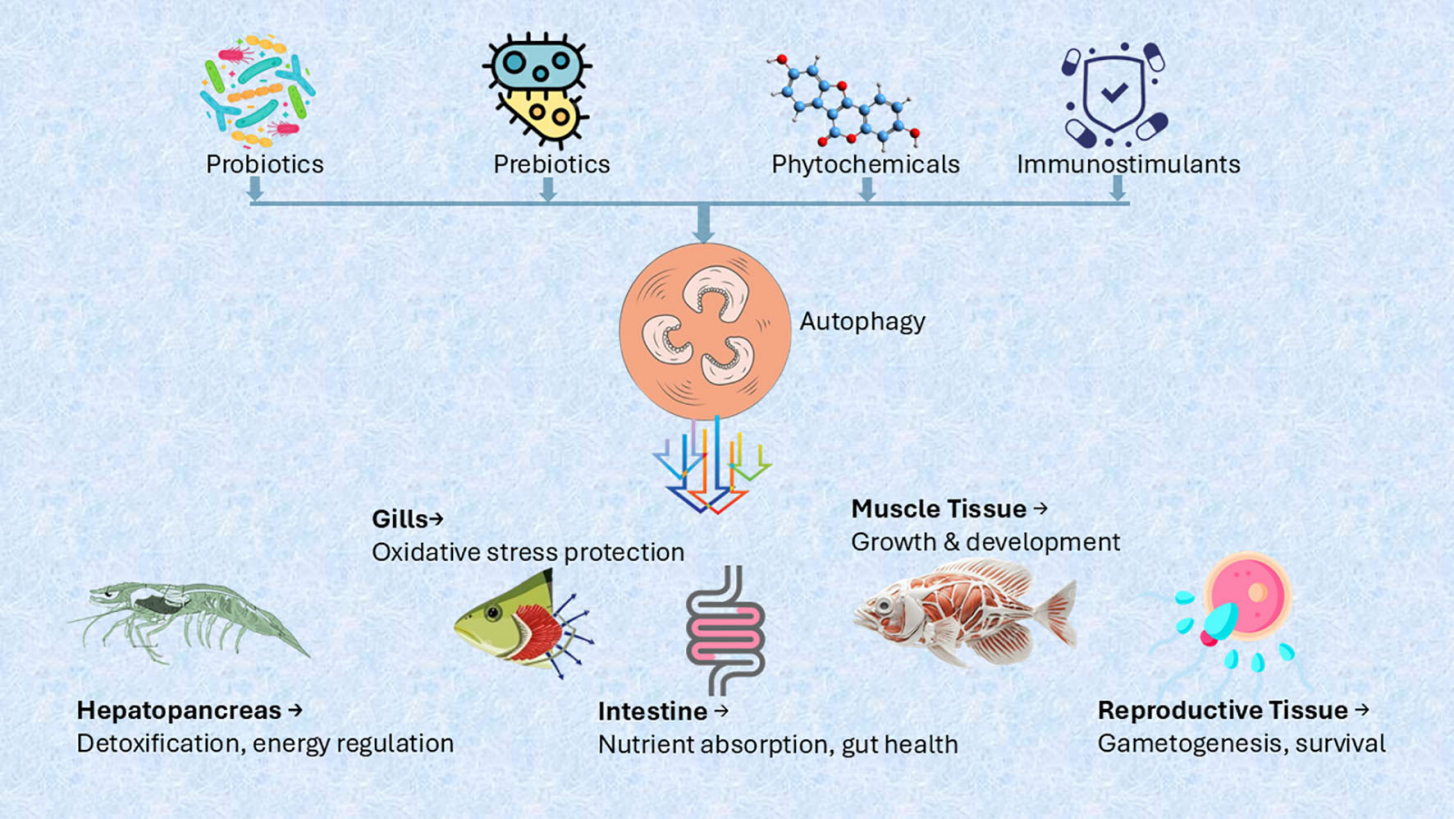
Probiotic-induced autophagy in aquaculture.

Additionally, probiotics enhance autophagic responses in intestinal epithelial cells, which is particularly beneficial for aquatic species prone to gastrointestinal infections. For instance, *Bifidobacterium breve* and *Lactobacillus plantarum* have been shown to induce autophagy in intestinal cells, strengthening their resilience under stress and protecting them from pathogen-induced damage (177). This underscores the potential of probiotics in improving gut health and mitigating enteric diseases in aquaculture (**Figure 3**).

Beyond gut health, autophagy-modulating probiotics offer significant applications in aquaculture by enhancing growth, disease resistance, and overall health. Bacillus species have been reported to improve growth performance and intestinal health through autophagy induction (178), a strategy that could benefit farmed fish and shellfish. Integrating probiotics into functional feeds may further reduce reliance on antibiotics, supporting more sustainable aquaculture practices. However, further research is needed to fully elucidate the mechanisms of probiotic-induced autophagy and optimize their application in aquaculture systems.

### 6.2 Genetic and epigenetic regulation

#### 6.2.1 CRISPR/Cas9 studies on autophagy in aquatic species

CRISPR/Cas9 genome-editing technology has emerged as a powerful tool to dissect autophagy-related pathways in aquatic organisms, offering precise gene knockout capabilities to explore gene function at both cellular and systemic levels (186). CRISPR/Cas9 genome editing has been effectively used to investigate autophagy-related genes (epg5, ambra1a, and ambra1b) in aquatic species specially zebrafish (*D. rerio*), a key aquatic model organism to study the roles of these genes in autophagy regulation, development, and disease modeling (187).

Expanding beyond zebrafish, CRISPR/Cas9 technology has also been applied to study autophagy in other aquatic species, such as rainbow trout (*Oncorhynchus mykiss*). In this case, researchers used CRISPR-Cas9 to generate knockout (KO) lines targeting autophagy-related genes like Lamp2A, observing significant physiological changes, including altered body size, organ indices, and liver proteome remodeling (188). Building on this, Wang et al. (189) highlighted the potential of CRISPR in freshwater fish species, such as fathead minnows, to study autophagy mechanisms (189). Their work demonstrated that HMGB1 paralogues (HMGB1a and HMGB1b) are crucial regulators of autophagy through modulation of LC3-II expressions. These findings not only provide valuable insights into autophagy regulation in teleosts but also suggest that CRISPR could be applied to study and potentially enhance autophagy in commercial fish species, offering a better understanding of cellular processes critical for aquaculture (189).

Similarly, beyond zebrafish, CRISPR/Cas9 has been applied in commercial aquaculture species like gibel carp (*Carassius gibelio*), with early studies targeting immune-related genes. Mou et al. (190) used CRISPR/Cas9 *in vivo* to knock out duplicated viperin genes (Cgviperin-A and Cgviperin-B), revealing their distinct roles in antiviral defense against *Carassius auratus* herpesvirus (CaHV). The study showed that these paralogues regulate immune and autophagy pathways, and their simultaneous disruption eliminated viral resistance, underscoring the potential of CRISPR in functional genomics and disease resilience in aquaculture (190). However, autophagy-targeted CRISPR studies in commercial fishes remain limited, highlighting a gap and potential for future research.

In addition to fish, CRISPR applications are also emerging in invertebrate aquaculture species, showing promising potential. For instance, in *Mulinia lateralis*, a commercially relevant bivalve, Wang et al. (191) used CRISPR/Cas9 to knockout Cfap206, uncovering its critical role in embryonic ciliogenesis and sperm flagellum formation (191). This study not only validates CRISPR-based functional genomics in marine invertebrates but also suggests its applicability in reproductive management strategies, further supporting its broader relevance across aquatic organisms (191). CRISPR/Cas9 has enhanced understanding of autophagy in aquatic models, uncovering key genetic interactions. It offers valuable insights into conserved molecular pathways, with future research set to deepen exploration of their interplay in aquatic species.

#### 6.2.2 RNAi-based interventions to control disease

RNAi-based interventions in aquatic animals represent a sophisticated approach to disease control by simultaneously targeting pathogens and modulating host autophagy and cell death pathways. Studies demonstrate that viral gene silencing via RNAi (e.g., targeting WSSV VP28 or YHV protease) not only reduces pathogen load but also restores beneficial autophagy flux and prevents excessive apoptosis in infected cells (192). For instance, dsRNA against WSSV rr2 in shrimp upregulates ATG5-dependent autophagy while reducing caspase-3 activation by 75%, illustrating RNAi’s dual role in pathogen clearance and cellular homeostasis maintenance (193, 194). The technology’s efficacy is further enhanced by advanced delivery systems like chitosan-rapamycin-dsRNA nanoparticles that combine gene silencing with autophagy induction, showing 3.2-fold greater tissue accumulation and prolonged antiviral effects compared to conventional methods (195, 196).

Emerging challenges include pathogen evolution of siRNA-resistant variants and tissue-specific differences in RNAi-autophagy coupling (197, 198), which are being addressed through multiplexed dsRNA designs and tissue-targeted delivery platforms (199). Future directions focus on integrating RNAi with CRISPR for synergistic gene editing (200), optimizing administration timing based on circadian rhythms of RNAi machinery activity (201), and engineering probiotic bacteria for gut-specific delivery (202). These developments position RNAi as a versatile tool that bridges pathogen-specific defense with fundamental cellular regulation, offering a comprehensive strategy for sustainable disease management in aquaculture systems facing increasing pathogen threats and environmental stressors.

## 7 Future directions and knowledge gaps

### 7.1 Unexplored molecular pathways

Although significant progress has been made in understanding autophagy and cell death mechanisms in aquatic animals, several molecular pathways remain underexplored. While approximately 90% of the core autophagy-related genes (ATGs) are conserved across eukaryotes, the specific functions and regulatory mechanisms of these genes in fish and other aquatic species remain largely unknown (203). The whole-genome duplication event that occurred 300–350 million years ago in teleost fish may have led to lineage-specific modifications in autophagy pathways, influencing crucial processes such as immune responses, tissue remodeling, and metabolic regulation (126). Therefore, comparative genomic and functional studies are essential to determine whether the autophagy-related gene expression and regulatory mechanisms observed in model organisms are applicable to aquatic species.

Additionally, selective autophagy pathways, such as mitophagy and chaperone-mediated autophagy (CMA), which are vital for cellular homeostasis, are still poorly understood in aquaculture species. The lysosome-autophagy system in fish has been widely used as a biomarker for aquatic environmental health (88). However, the molecular mechanisms linking lysosomal integrity, toxicant accumulation, and autophagy dysfunction remain unclear (125). While transgenic zebrafish and zebrafish-derived cell lines have proven to be valuable models for studying autophagy regulation, research on other fish species, especially those of aquacultural importance, remains limited. Expanding studies to include a broader range of fish species could provide deeper insights into species-specific autophagy responses and their implications for growth, immunity, and stress adaptation.

Despite advancements in understanding autophagy in fish, several molecular pathways, particularly those related to disease response and immune regulation, remain unexplored. One significant gap is understanding the spatiotemporal characteristics of autophagy during microbial infections. For example, in nervous necrosis virus (NNV) infection, autophagy expression exhibits distinct temporal and spatial patterns, but its precise molecular role and regulatory mechanisms remain undetermined (204). Further investigation is needed to determine whether autophagy serves a protective function or facilitates viral replication. Addressing this knowledge gap could offer critical insights into host-pathogen interactions and help develop targeted therapeutic strategies for viral diseases in aquaculture.

Chaperone-mediated autophagy (CMA), long thought to be restricted to tetrapods due to the apparent absence of LAMP2A, a key component of this pathway, in other species (205), has recently been shown to exist in certain fish species. These species possess sequences with high homology to mammalian LAMP2A, suggesting that CMA may have an evolutionary role in fish autophagy regulation (206). This discovery challenges previous assumptions and highlights a critical gap in our understanding of CMA’s function in fish. Further research is required to explore its molecular mechanisms, regulatory pathways, and potential implications for fish health, immunity, and adaptation, especially in response to environmental and pathogenic stressors.

Currently, only few studies has explored the relationship between Ostreid herpesvirus 1 (OsHV-1) infection and autophagy in the hemolymph and mantle of Pacific oysters (35). However, the broader implications of autophagy in the immune responses of other aquatic invertebrates, such as shrimp, mussels, and other mollusks, remain largely unexplored. More studies are needed to elucidate the molecular mechanisms underlying autophagy-mediated antiviral defense in invertebrates. These insights could provide novel strategies for disease management in aquaculture (10, 58)However, the molecular mechanisms by which ATG proteins modulate innate immunity in aquatic animals are still largely unknown. Although studies have demonstrated the interplay between viral infections and autophagy in aquatic species, the exact role of ATG proteins in antiviral defense mechanisms remains unclear (10). Addressing this research gap could provide critical insights into immune regulation in aquatic organisms and contribute to the development of novel disease management strategies in aquaculture.

### 7.2 Potential for disease control in aquaculture

Although research on autophagy in fish is still in its early stages, emerging evidence suggests that this process holds significant potential for disease control in aquaculture. Autophagy has been shown to modulate immune responses by degrading intracellular pathogens and regulating inflammation, highlighting its importance in host defense (207). While transgenic zebrafish and zebrafish-derived cell lines have been widely used to study autophagy regulation, its role in disease resistance among commercially important fish species remains largely unexplored (88).

The lysosome-autophagy system, commonly employed as a biomarker for aquatic ecosystem health, also plays a critical role in pathogen defense by modulating immune responses at the cellular level (88). Understanding how autophagy influences pathogen-host interactions in fish could lead to novel disease management strategies, including the development of autophagy-targeting therapeutics and dietary interventions to enhance immunity (207, 208). Additionally, nutrient deficiency-induced autophagy has been shown to mitigate oxidative stress, potentially reducing susceptibility to infections in aquaculture species (88).

Future research should focus on elucidating the molecular mechanisms through which autophagy interacts with fish immune pathways, paving the way for innovative approaches to disease prevention and control. Exploring autophagy’s regulatory role in immune modulation could contribute to the development of targeted interventions aimed at enhancing disease resistance and overall fish health. Autophagy modulators such as probiotics, plant-derived compounds, and immune-stimulants warrant further investigation to optimize their application in aquaculture. Additionally, RNA interference (RNAi)-based strategies show promise for pathogen control by modulating host autophagy and cell death pathways. However, challenges related to cost, environmental safety, and delivery methods must be addressed before these approaches can be widely implemented in aquaculture.

### 7.3 Need for high-throughput studies and multi-omics approaches

High-throughput screening methods, when combined with CRISPR/Cas9, offer a powerful approach to systematically dissect autophagy pathways and identify novel regulators or therapeutic targets (209–211). The zebrafish epg5 mutant line, for instance, serves as a model for high-throughput drug screening to identify compounds that can modulate autophagy flux without disrupting its essential functions (187). Additionally, phenotypic analysis of ambra1a and ambra1b mutants highlights the potential of multi-omics approaches- such as transcriptomics and proteomics-in uncovering compensatory mechanisms and the sub-functionalization of paralogous genes (212).

Future research should expand the use of CRISPR/Cas9 to target additional autophagy-related genes in aquatic models for comprehensive functional analysis. Integrating genomic, transcriptomic, and proteomic data will enhance mapping of autophagy networks and their crosstalk with other pathways. High-throughput platforms can also be employed to screen autophagy-modulating drugs for conditions like neurodegenerative diseases and cancer (213). Combining CRISPR/Cas9 with multi-omics and advanced bioinformatics will help uncover conserved and species-specific autophagy mechanisms and advance its therapeutic exploration in aquatic and mammalian systems (213, 214).
